# Plants Under Stress: Exploring Physiological and Molecular Responses to Nitrogen and Phosphorus Deficiency

**DOI:** 10.3390/plants13223144

**Published:** 2024-11-08

**Authors:** Swarup Mishra, Hannah Levengood, Jinping Fan, Cankui Zhang

**Affiliations:** Department of Agronomy and Center for Plant Biology, Purdue University, West Lafayette, IN 47907, USA; hjs8zu@virginia.edu (S.M.); hlevengo@purdue.edu (H.L.); jinpingfan@neau.edu.cn (J.F.)

**Keywords:** nitrogen, phosphorus, deficiency, local signaling, long distance signaling

## Abstract

Nitrogen (N) and phosphorus (P) are essential mineral macronutrients critical for plant structure and function. Both contribute to processes ranging from cellular integrity to signal transduction. Since plants require these nutrients in high concentrations, replenishing them in soil often involves chemical fertilizers. However, the main source of P, rock phosphate, is non-renewable and in decline. N, second only to carbon, oxygen, and hydrogen in plant requirements, is vital for synthesizing proteins, nucleic acids, and plant pigments. Although N is available to plants through biological fixation or fertilizer application, the frequent application of N is not a sustainable solution due to environmental concerns like groundwater contamination and eutrophication. Plants have developed sophisticated mechanisms to adapt to nutrient deficiencies, such as changes in root architecture, local signaling, and long-distance signaling through the phloem. A dual deficiency of N and P is common in the field. In addition to individual N and P deficiency responses, this review also highlights some of the most recent discoveries in the responses of plants to the combined N and P deficiencies. Understanding the molecular and physiological responses in plants to mineral deficiency will help implement strategies to produce plants with high mineral use efficiency, leading to the reduced application of fertilizers, decreased mineral runoff, and improved environment.

## 1. Introduction

Nitrogen (N) and phosphorus (P) are two essential macro-mineral nutrients that play critical structural and physiological roles in plants. N is required in the largest quantities in plants after carbon, oxygen, and hydrogen. It serves as the building block for the biosynthesis of proteins, nucleic acids, and pigments and plays a central role in plant metabolism, growth, and development [1]. N can be fixed biologically or atmospherically. Biological N fixation occurs in legumes, converting nitrogen to ammonium (NH_4_^+^), which is then used by plants [2]. Atmospheric N fixation occurs when N is converted to nitric acid via lightning, which dissociates into nitrate (NO_3_^−^) [2]. Both NH_4_^+^ and NO_3_^−^ are plant-available forms. They enter a biogeochemical cycle, eventually returning to the atmosphere as N_2_ after several rounds of conversion between organic and inorganic forms [2]. P, another essential macro-mineral, is crucial for several structural and functional processes. While N drives protein and nucleic acid biosynthesis, P is a key component of nucleotides like ATP, which are vital for energy transfer and storage in cells [3,4]. P also forms part of phospholipids, essential for cell membranes, and regulates enzymes in metabolic pathways. Furthermore, P plays a pivotal role in root development, flowering, and fruiting, contributing to overall plant vigor. Unlike N, P is not as readily cycled through the atmosphere; it is primarily derived from soil minerals and organic matter.

The uptake, translocation, and remobilization of NH_4_^+^ and NO_3_^−^ occur through membrane proteins on root surfaces. Once inside the plant, NO_3_^−^ is reduced to nitrite (NO_2_^−^) by nitrate reductase in the cytoplasm and then to NH_4_^+^ by nitrite reductase in plastids, with further conversion into glutamine via the GS/GOGAT (glutamine synthetase/glutamate-2-oxoglutarate aminotransferase) cycle, followed by the production of other amino acids [5]. Similarly, plants absorb P primarily as phosphate anions (Pi) via membrane transporters [4]. Different from N, the absorbed Pi can be directly assimilated to plant metabolism without being converted to other forms of P inside the plants [4,6,7].

Research shows that most soils worldwide are deficient in N and P, which severely impacts plant and crop yields [8]. NH_4_^+^ is often immobilized by cation exchange sites or converted by microorganisms to NO_3_^−^ via nitrate reductase. Additionally, N can become unavailable to plants due to leaching, denitrification, nitrification, and volatilization [9]. On the other hand, plants struggle with P acquisition due to its limited availability, even in fertile soils [10]. P often forms insoluble complexes with organic matter or with iron, aluminum, and calcium [11]. Therefore, supplementing soils for N and P with chemical fertilizers has become a regular practice in intensive agricultural systems. However, the overuse of fertilizers is not sustainable and poses environmental and health risks, such as groundwater contamination, eutrophication, biodiversity loss, soil acidification, atmospheric pollution, and global warming due to greenhouse gases [12,13]. Climate change and pollution further threaten crop yields and human health [14,15].

Given these challenges, it is crucial to understand plant responses under mineral-deficient conditions to develop strategies for crops with improved mineral-use efficiency [16]. As sessile organisms, plants have evolved unique mechanisms to respond and adapt to nutrient stresses. Under N or P deficiency, a series of alterations occur in plants, including the modification of their root architecture and the increase in the expression of root mineral transporters [1,3]. However, the responses to N and P deficiencies are distinct. This review focuses on morphogenic, physiological, and molecular responses to N or P deficiency in plants. Additionally, we will discuss recent studies on combined N and P deficiencies, which are common in the field, as well as the interactions between these two nutrients. In particular, we will highlight the role of shoot-to-root long-distance signaling, which plays a critical role in regulating root responses to low N or P availability. This review not only covers some of the recent findings in the areas but also covers important discoveries made in the last few decades. One unique aspect of this paper is its inclusion of both the molecular and physiological responses to N and P within a single manuscript. This comprehensive approach enables a clearer comparison of these two essential macro-nutrients, particularly their interactions.

### 1.1. Properties of P and Plant Responses to P Deficiency

Soil P is the primary source that plants depend on to meet their nutritional requirements. It exists in two important chemical forms: inorganic (Pi) and organic (Po). Pi accounts for 35–70% of the total soil P pool [17]. P release involves the weathering of primary minerals (apatites, strengite, and variscite), which is a very slow process, and that of secondary minerals (calcium, iron, and aluminum phosphates), which vary in their dissolution rates depending on the soil pH and size of the mineral particles [18]. The availability of orthophosphates also varies with the pH of the soil solution, with H_3_PO_4_^−^ dominating under low pH conditions and PO_4_^3−^ dominating under high pH [4]. H_2_PO_4_^−^ and HPO_4_^2–^ are the two main orthophosphates absorbed by plants, with the preferential absorption of H_2_PO_4_^−^ within a pH range of 4.5–5.0 [4].

P deficiency results in a number of symptoms in plants, which are initially observed in older or mature leaves due to the mobility of the nutrients in the phloem. Early signs include dark to blue-green leaf coloration, which can progress to purple in severe cases. Several important plant developmental processes also require P for proper function. For example, since P is integral to cell division [19], its deficiency can lead to the delayed initiation of new leaves, an increased shoot-to-root ratio, and overall stunted growth [20,21]. Additionally, since P is present in high quantities as phytic acid in seeds and grains, its limitation leads to delayed maturity and poor fruit and seed development.

The following sections provide a brief review of the biochemical, physiological, and molecular responses of plants to P deficiency.

### 1.2. Biochemical and Physiological Responses to P Deficiency

P is vital for multiple biochemical processes, including lipid metabolism, signal transduction, energy storage and mobilization, photosynthesis, and respiration. As a result, low P levels restrict plant growth and reproduction. To cope with deficiency, plants have developed an array of developmental and metabolic responses [16,22]. These responses are defined as plant starvation responses (PSRs), which occur when the concentration of P drops below 10 µM in the plant tissue [23]. Since inorganic P (Pi) is mobile in nature and is directly acquired by the roots, it serves as the primary signaling molecule in regulating PSRs. Additionally, alterations in cellular Pi contents trigger the biosynthesis of various components, including miRNAs, mRNAs, sugars, hormones, transcription factors, peptides, P-containing metabolites, and reactive oxygen species (ROS). These components collectively elicit a range of diverse responses in the plant [3,16]. Importantly, PSRs are also influenced by other nutrients and hormones, suggesting that P is involved in crosstalk at multiple levels.

Besides the dark/purple coloring of mature leaves, other symptoms of Pi in the cytosol include impaired photosynthesis, stunted growth, increased branching, weakening of the stems and leaves, compromised pollination, and reduced flowering rates. P deficiency has a severe effect on yield parameters, such as grain quality, grain filling, and tillering. It also detrimentally affects the normal opening and closing of the stomata, increases the senescence of older leaves, and results in the remobilization of stored Pi to younger foliage. At the cellular level, symptoms include increased sugar concentrations and the transfer of vacuolar Pi to the cytoplasm [24].

PSRs can be categorized into local and systemic responses. Local responses improve root soil exploration in the rhizosphere by altering the Root System Architecture (RSA), while systemic responses, also known as long-distance responses, depend on cellular Pi concentrations and contribute to improved uptake, allocation, mobilization, and assimilation [25]. Plants exhibit notable physiological responses during or after exposure to P-deficient conditions, and these responses can be attributed to variations in plant metabolism. For example, anthocyanin levels increase in the leaves, which help to protect nucleic acids and chloroplasts from harmful UV light [26].

One of the most prominent PSRs is root-tip inhibition and remodeling of the RSA. RSA remodeling involves a reduction in primary root growth and an increase in root branching and lateral root growth. For instance, in rice, the actin-binding protein *RMD1* is induced by low Pi conditions, causing the roots to take up more nutrients from the topsoil layer by controlling the crown root angle [27]. Similarly, the inhibition of the root tip is regulated by two ferroxidases, LPR1 and LPR2 (LOW PHOSPHATE ROOT), which are further dependent on the endoplasmic reticulum-located PHOSPHATE DEFICIENCY RESPONSE2 (PDR2), under low P conditions. These enzymes result in an overall increase in Fe^3+^ ions in the apoplast following their oxidation from Fe^2+^, with a buildup of ROS and callose in the root meristem [28,29]. In addition, the SENSITIVE TO PROTON RHIZOTOXICITY1 (STOP1) transcription factor also increases the concentration of Fe^3+^ ions by activating ALUMINUM ACTIVATED MALATE TRANSPORTER1 (ALMT1) under P deficiency, resulting in increased generation of malate ions [30]. This causes a reduction in intercellular symplastic movement of SHR (SHORT ROOT) and leads to its subsequent accumulation [31]. Since SHR is involved in meristem maintenance, impaired function leads to premature cell differentiation and the suspension of mitotic activity [30,32]. Inhibition of the root tip therefore requires extracellular Pi-sensing and is dependent on local Pi in the rhizosphere [33].

Another striking feature of plant responses to low Pi is the increased growth of lateral roots and the development of root hairs. Low Pi causes a reduction in root cell elongation due to an increase in root density and the development of cluster roots in some species in the *Casuarinaceae*, *Fabaceae*, *Myricaceae*, and *Proteaceae* families. This results in a marked decrease in primary root growth, with a shift in meristematic activity towards lateral roots [24,34].

Various plant hormones, including auxins, gibberellins, ethylene, and strigolactones, mediate this phenomenon, all of which are involved in multiple developmental processes. Pi deficiency also stabilizes EIN3 (ETHYLENE INSENSITIVE-3), which binds to the promoter of RSL4 (ROOT HAIR DEFECTIVE SIX-LIKE4), promoting root hair development [35,36]. Low Pi also causes increased auxin-sensitivity and therefore, increased responsiveness to exogenously applied auxin. This also enhances the activity of several auxin-related genes, including TRANSPORT INHIBITOR RESPONSE1 (TIR1), ARF19 AUXIN RESPONSE FACTOR19 (ARF19), and ARABIDOPSIS RECEPTOR KINASE2UBOX (ARK2) [37].

Additionally, root hair elongation was also found to be regulated by a network consisting of ARF19, RHD SIX-LIKE 2 and 4 (RSL2, and RSL4) under low Pi conditions [38]. Like auxin and ethylene, gibberellins also control root hair development during low Pi conditions. GA levels drop during Pi deficiency, causing an increase in DELLA proteins [39], while strigolactones play an important role in root hair development by inhibiting shoot branching. Concurrently, a GR24-mediated reduction in lateral root formation was observed in Strigolactone biosynthesis mutants (*max3-11* and *max4-1*), causing elongation of the root hairs [40]. HYPERSENSITIVITY TO LOW PHOSPHATE-ELICITED PRIMARY ROOT SHORTENING1 HOMOLOG2 (HHO2) is a MYB62-related transcription factor that is involved in the harnessing and signal relay and subsequent lateral root production under low Pi [41]. The overall increase in lateral roots and root hairs is therefore the result of an increase in the root/shoot ratio, which benefits the acquisition of Pi from the root rhizosphere environment [42].

To scavenge P from the soil rhizosphere, plants modify the immediate biochemical environment around the roots by releasing organic anions (malate, citrate, and oxalate), phenolic acids, enzymes, and other compounds. These chemical compounds facilitate the solubilization of Pi from insoluble P reserves [43]. Some plants also resort to mycorrhizal associations with certain symbiotic fungi [44]. These plants also have the unique ability to secrete enzymes (phosphatases and organic acids), which solubilize unavailable forms of Pi and make them more available for plant uptake.

Purple acid phosphatases (PAPs) are an important group of metallohydrolases that enhance the uptake of Pi by cleaving Pi from exogenous Pi mono- or di-esters such as glucose-6-phosphate, DNA, RNA, ATP, and glycerate [16]. They also induce the activation of high-affinity transporters and assist in the translocation of Pi from senescing leaves to younger leaves [45]. Under Pi-deficient conditions, intracellular and secretory PAPs are upregulated to assimilate Pi. These PAPs include AtPAP17, AtPAP11, AtPAP5, AtPAP14, AtPAP22, AtPAP23, AtPAP25, and AtPAP26 [23].

Additional physiological responses to Pi deficiency include a build-up of starch and increased carbohydrate partitioning. This results in an increased accumulation of 3-phosphoglycerate (3-PGA), which induces the AGPase enzyme, a component of the starch biosynthesis pathway. APGase is vulnerable to inhibition upon binding of Pi to its active site [23], so when Pi is decreased below a threshold level, APGase activity and starch biosynthesis are increased [23]. Several studies on gene expression have revealed the importance of sucrose as an important regulator in mediating the expression of PSI genes [46]. Although the exact mechanisms of sucrose-signaling influencing Pi starvation-induced gene expression remain unknown, sucrose is thought to be involved in cross-talk with the Pi-deficiency responses in plants [47].

Active lipid remodeling also takes place under low Pi. Lipids are energetically expensive to synthesize. Therefore, to reduce the amount of energy required, plants replace phospholipids, a major membrane lipid in the plasma membranes, with other lipids devoid of P, such as galactolipids like sulfoquinovosyldiacylglycerol (SQDG) and digalactosyldiacylglycerol (DGDG) [48]. *Pho1* mutants exhibit enhanced levels of DGDG and SQDG in their leaves and decreased levels of phospholipids [49]. Mass spectrometry and RNA-Seq analyses pointed towards alterations in acyl-CoA pool and ER-derived precursors and therefore diverted the flux towards triacylglycerol synthesis [50]. Low Pi conditions were also found to upregulate the MGD2 (Monogalactosyldiacylglycerol Synthase) /3-DGD-2 pathway, which causes membrane lipid remodeling in Arabidopsis (*Arabidopsis thaliana*) [51].

### 1.3. Local and Systemic Signaling Events in Plants During P Deficiency

As sessile organisms, plants have unique mechanisms that allow them to sense deficiencies and relay the signals in order to improve the use of a particular nutrient. As a result, plants can sense the limited availability of P and reprogram their transcriptional machinery to maintain homeostasis. Pi acquisition through organelles, including the chloroplasts, mitochondria, Golgi apparatus, and cell membrane, occurs via a suite of different transporter proteins, PHOSPHATE TRANSPORTER 1/2/3/4/5(*PHT1*, *PHT2*, *PHT3*, *PHT4*, and *PHT5*) in Arabidopsis [52,53,54,55,56]. The long-distance transport of Pi from the roots to shoots is facilitated by *PHOSPHATE1* (*PHO1*), *PHOSPHATE2* (*PHO2*), and *PHOSPHATE TRANSPORTER TRAFFIC FACILITATOR1* (*PHF1*), the latter assisting in the exit of Pi transporters from the ER and targeting of the plasma membrane. Earlier research suggests that mutations in these genes result in deviations in shoot and/or root concentrations of Pi [57,58,59]. As previously mentioned, signaling events during Pi deficiency can be grouped into two types depending on the perception of signals and assimilation: local and systemic, which are detailed below.

Pi deficiency is primarily sensed at the root tips, where low Pi zones lead to a decrease in Pi concentrations in the root cell apoplasm. This stimulates a primary response via uncharacterized sensors located on the plasma membrane. The internal Pi status is a more important driver of Pi starvation responses than external fluctuations [60]. Divalent calcium ions, inositol phosphates (IPs), and ROS trigger local Pi sensing and signaling. For example, inositol 1,4,5-triphosphate (IP3), a phosphorylated inositol, regulates Ca^2+^ release, indicating crosstalk among these molecules. Mutations in genes encoding vacuolar Ca^2+^/H^+^ exchangers result in compromised inositol polyphosphate kinase activity, leading to an accumulation of Pi in the shoots [61]. Additionally, inositol phosphate kinase 1 (IPK1) catalyzes the last step of phytate biosynthesis, and impaired activity causes an overall accumulation in intermediates (IP4 and IP5), thus altering Pi homeostasis [62].

ROS also plays an important role in local Pi signaling, as their levels increase in the roots and trigger the upregulation of various Pi-responsive genes. A mutation in ROOTHAIR DEFECTIVE2 (RHD2), which codes NADPH oxidase, was found to impair ROS production and prevent the induction of these genes [63]. Several phytohormones are also known to crosstalk and influence Pi-responsive sensing and signaling. Auxin, ethylene, and strigolactones act as positive mediators of Pi-starvation signaling, while gibberellic acid, cytokinins, and abscisic acid act as negative regulators [64]. Except for GAs and CKs, which decrease, other phytohormones are typically induced during Pi deficiency [60]. These hormones mostly act to reprogram the RSA, improving the plant’s ability to acquire Pi from the topsoil. Systemic Pi signaling remains the major component of plant responses during Pi starvation. This includes systemic root-to-shoot and shoot-to-root signaling, as well as shoot apical meristem tissue signaling [65]. Most systemic responses to Pi deficiency are manifested during leaf development, flowering time regulation, shoot meristem activity, root growth alterations, Pi sensing and recovery, recycling, and homeostasis [60,65].

Transcriptionally, most promoters of genes involved in Pi responses, which act as targets of transcription factors, are enriched with the P1BS motif [25]. PHOSPHATE STARVATION RESPONSE1 (*PHR1*) is the main transcription factor that mediates most downstream responses to Pi starvation [66]. It encodes a transcription factor harboring a coiled-coil (CC) domain within the GARP family of R2R3 MYB DOMAIN PROTEIN (MYB). Similarly, the important transcription factors PHR1-LIKE1 (PHL1) and PHL2 also belong to the same family [67]. Loss-of-function mutation of *PHR1* results in compromised expression of the PSI genes, including those controlling the shoot–root ratio and anthocyanin accumulation [68]. Major players in the root–shoot pathway of signal transduction include *PHO1*, cytokinins, and strigolactones. *PHO1* is also involved in Pi loading via the xylem from the roots to the shoot, with mutations in this gene causing a reduction in Pi levels in the shoot [57]. Supplementing the growth medium with cytokinins represses PSI genes, suggesting a role in the negative regulation of root–shoot signal transduction under low Pi conditions [57]. Meanwhile, *ahk3* mutants exhibit reduced sensitivity to cytokinins during the repression of PSI genes [64], while strigolactone biosynthesis is positively induced during Pi deficiency, particularly in the xylem sap, which suggests a role in root–shoot signaling during low Pi [69].

Just like the xylem, the phloem also functions as an important channel for the conduit of low Pi-mediated signals. Major shoot–root signal molecules include miRNAs, hormones, long non-coding RNAs, mRNAs, proteins, and sugars [3]. MicroRNA 399 (*MiR399*) is induced during Pi starvation and is involved in the decrease in PHO2 transcript levels, which encode an E2 conjugase. It has also been reported that PHO2 is involved in the ubiquitination of downstream Pi transporters such as *PHT1;8* and *PHT1;9*, as shown in mutational studies on *PHO2* [70]. Moreover, *pho2* plants show enhanced levels of *PHO1*, an important factor in Pi transport from the roots to the shoot, suggesting that PHO2 is a negative regulator of PSI and that miRNA399 is required for the maintenance of homeostasis within the plant [70]. In addition, PHO2 is also responsible for the degradation of PHO1, as shown by the partial colocalization of both genes in a tobacco system [71]. *PHF1* is thought to be a downstream target of PHO2, aiding membrane localization of PHT1;1 in the plasma membrane [59]. Proteins with SPX domains in their N-terminal region are also thought to be involved in Pi sensing and signaling in plants [72]. SPX proteins are further classified into EXS (ERD/XPR1/SYG1), MFS (Major Facilitator Superfamily), and RING (Really Interesting New Gene) types [72,73]. PHO1 encodes an SPX-EXS protein involved in Pi export and localization, while expression of the EXS domain in the *pho1* roots results in an overall improvement in shoot growth, suggesting plausible root-to-shoot communication [74].

**Figure 1:**
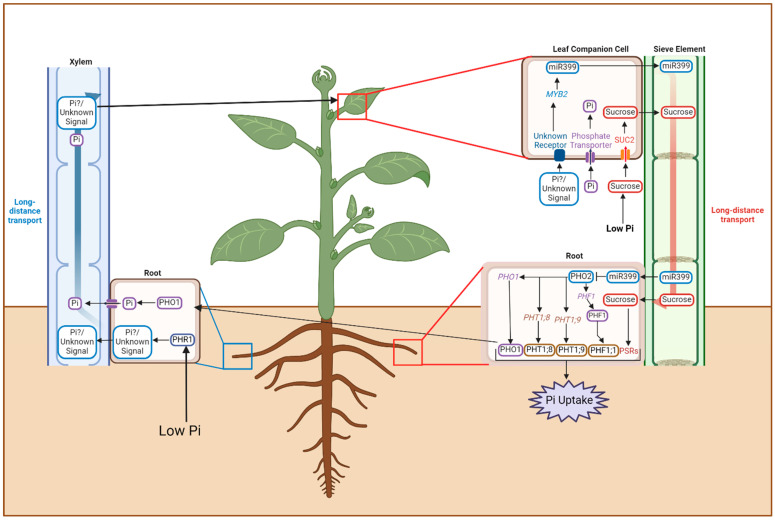
Systemic signaling responses to Pi deficiency in plants. Root-to-shoot signaling is via the xylem, and shoot-to-root signaling is via the phloem. PHO1: phosphate 1; PHR1: phosphate response 1; PHF: phosphate transporter traffic facilitator 1; MYB2: myb domain protein 2; SUC2: sucrose transporter 2; PHT: phosphate transporter; miR399: MicroRNA399. This image was generated using biorender.com.

Sucrose reportedly also acts as a shoot-to-root systemic signaling molecule, resulting in upregulation of several PSRs in plants, as shown in girdling experiments [75]. During Pi deficiency, there is an accumulation of sucrose in the shoots, which suggests close involvement in the regulation of Pi deficiency signaling [47]. SUCROSE TRANSPORTER2 (SUC2) functions as an essential sucrose transporter, and its knock-out results in the reduction in the expression of PSI genes [46]. The efficient function of this transporter protein depends on the establishment of an electrochemical gradient, which is facilitated by ATP. Following activation, the transporters pump sucrose into the system, which is utilized for energy generation. Induction of genes involved in de novo synthesis and sucrose oxidation, such as *SUSY2* (*SUCROSE SYNTHASE 2*), *GAPC-2* (*GLYCERALDEHYDE-3-PHOSPHATE DEHYDROGENASE*), and *PPase* (*PYROPHOSPHATASE*), have also been reported during Pi deficiency [76]. Pumping in higher concentrations of sucrose helps enhance the root biochemistry, improving P acquisition, increasing the activity of Pi transporters, inducing the release of acid phosphatases for increased P scavenging from the soil, and improving P assimilation in the plant [47]. Systemic signaling in response to P deficiency is summarized in Figure 1.

### 1.4. Epigenetic and Genetic Alterations During Pi Deficiency

Stable and heritable alterations in the expression and function of genes without alterations in the genome or DNA are referred to as epigenetic modifications. Such modifications include but are not limited to DNA methylation, histone acetylation, genomic imprinting, and gene silencing of transposable and repetitive elements. Epigenetic states are highly versatile and subject to remodeling due to environmental fluctuations [77]. DNA methylation is a prominent epigenetic modification strategy. Low Pi conditions induce widespread changes in DNA methylation patterns, which manifest as various morphological and physiological alterations. By analyzing whole-genome differential methylation patterns, DNA methyltransferases (DNMT) were found to cause changes in DNA methylation, resulting in the regulation of gene expression patterns [77]. However, these changes are directly controlled by the master transcription factor PHR1. This study also revealed that *SPX2* is hypomethylated by MET1 (METHYL TRANSFERASE 1), further preventing the inactivation of PHR [77]. Similarly, hypomethylation of *miR827* suggested negative regulation of *NLA* (NITROGEN LIMITATION ADAPTATION) transcript levels, which relieves negative regulation of *PHOSPHATE TRANSPORTER 1* (*PHT1*) [78]. Additionally, upstream sequences flanking low-Pi responsive genes were also found to be the subject of DNA methylation [78], and ARP6 (ACTIN-RELATED PROTEIN6), which forms part of the SWR1 chromatin remodeling complex causing H2A.Z accumulation, failed to function under low-Pi conditions [79]. Despite these findings, more studies into the mechanisms of epigenetic modifications are required to fully understand these signaling pathways.

The genetic regulation of low-Pi signaling is relatively complex. Key genes involved or induced during this process have been determined by genetic analyses, such as genome-wide microarrays, suppression subtractive hybridization, deep-sequencing assays, T-DNA knock-out, RNAi, RNA-Seq, and other studies involving mutants. Genes are largely involved in Pi acquisition, mobilization, signaling, and assimilation during growth, development, and metabolism [23], while several cis-acting sequences were found to influence the sensitivity of PSI genes, such as PIBS, MBS (MYB-acting site), and W-Box elements, thereby mediating Pi-starvation responses [26,66,80]. P1BS motifs (GNATATNC) are found in the promoters of PSR genes and near the mycorrhiza transcription factor binding sequence (MYCS) in arbuscular mycorrhizae (AM) fungi. P1BS motif is reportedly involved in tolerance to Pi starvation in crop plants such as soybean [81].

As mentioned above, PSRs are dependent on a plethora of different transcription factors, some of which are induced irrespective of Pi starvation, such as PHR1 and PHR1-Like in Arabidopsis and OsPHR1 and OsPHR2 in rice [26,66,82]. Transcription factors specific to PSRs include MYB62, ZAT6, WRKY6, WRKY75, bHLH32, and OsPTF1. Prolonged Pi-deprivation-induced expression of MYB62, ZAT6, and HRS1 have also been reported [83], while WRKY6, WRKY45, and WRKY75 were found to bind to W-Box regions (TTGACCT/T), which are enriched in promoters of Pi transporters [83,84]. Similarly, PRD and bHLH transcription factors were found to mediate PSI by binding to respective promoter motifs [85,86] while induction of other common bHLH transcription factors, such as rice OsPTF1, soybean GmPTF1, and maize ZmPTF1, was also observed in Pi-deficient roots [81,87].

## 2. Nitrogen

### 2.1. Properties of N and Plant Responses to N Deficiency

N is an inert element that is highly abundant and accounts for 78% of the atmosphere. The highly inert bond between two N atoms requires a lot of energy (941 kJ/mol) and is difficult to break into reactive N (N_r_), rendering the element difficult for plant utilization [2]. N_r_ consists of two main reduced forms: ammonium (NH_4_^+^) and ammonia (NH_3_). Several oxidized forms also exist, such as nitrate (NO_3_^–^), nitrite (NO_2_^–^), nitrous oxide (N_2_O), nitric oxide (NO) and nitrogen dioxide (NO_2_). Organic forms include urea and amines. N is consumed by plants in the form of NH_4_^+^ or NO_3_^–^ ions, which are later converted to amino acids, proteins, and DNA [1]. N deficiency limits a plant’s ability to perform normal growth and development. Given the importance of N to plants, various alterations in plant physiology, metabolism, development, signaling, and genetics in response to N deficiency have been summarized in the following sections.

### 2.2. Biochemical and Physiological Responses in Plants During N Deficiency

Low NO_3_^−^ results in an overall reduction in the activity of several biochemical processes involving N, including nitrate reductase (NR), nitrite reductase (NiR), phosphoenol pyruvate carboxylase (PEPCase), glutamine synthetase (GS), and the concentrations of organic acids such as oxo-glutarate, citrate, isocitrate, and malate. Additionally, starch synthesis exhibits an inverse relationship with shoot nitrate contents [85,86]. To sustain metabolism, plants salvage N from nucleic acids and enzymes, in turn mimicking the leaf senescence phenotype [88]. The sugar/N ratio also increases with reductions in N levels, thereby accelerating this process of senescence [89]. Other effects of low NO_3_^−^ on plant metabolism include a reduction in ethylene hormone and downregulation of ACS and ACO enzyme activity [90].

Plants growing in environments with differing NO_3_^−^ availabilities restructure their RSA in diverse ways. For example, the total root length exhibits a dose-dependent increase under mild N deficiencies and reductions under severe deficiency [91]. In general, the root system possesses two main responses: a local response, with lateral root elongation in NO_3_^−^ patches only, and a systemic response, during which the N status of the entire root system is signaled to the shoots, relaying a signal to curb lateral root growth under uniformly high N levels [92,93,94]. The systemic repression of lateral root growth was found to be highest when the roots were under uniformly high NO_3_^−^ conditions compared to compartmentalization of high NO_3_^−^ in a split-root setup [92]. In another study, lateral root growth in *nrt1.1* mutants failed to respond with normal elongation, suggesting a role of *nrt1.1* in lateral root elongation [95] NPF6.3 and NRT2.1 were also found to play a role in the basipetal transport of auxin from lateral root primordial (LRP) cells, thereby reducing auxin accumulation around these cells [96]. Auxin transport was hindered under high levels of NO_3_^−^ [96].

In addition to transcriptional control of NRT2.1 expression, protein levels were also discovered to be downregulated by NO_3_^−^ in LRP cells but not the entire root system, thereby reducing lateral root initiation [97]. The reduced accumulation of NO_3_^−^ also reduced basipetal transport of auxin, in turn causing an increase in auxin in LRP cells [97]. The pattern of lateral root growth is also dependent on the heterogeneity of NO_3_^−^ availability. During low NO_3_^−^ conditions, NO_3_^−^ poor patches experience a reduction in lateral root development, while in NO_3_^−^ rich patches, lateral root development is triggered. These processes were found to be orchestrated by *NRT1.1* [94]. The NLP-NIGT1-NRT2.1 transcriptional cascade also explains the uptake of NO_3_^−^ from NO_3_^−^-rich patches. NIN-like protein (NLP) reportedly activates *NRT2.1* and *NIGT1* when supplied with NO_3_^−^, thereby resulting in an initial increase in NO_3_^−^ uptake. However, NIGT1 subsequently represses *NRT2.1*, thereby attenuating NO_3_^−^ uptake when NO_3_^−^ concentrations reach an optimum level [98].

Another important component of lateral root development is RABIDOPSIS NITRATE REGULATED 1 (*ANR1*), a MADS-BOX transcription factor. Overexpression of this results in increased lateral root growth during exposure to high NO_3_^−^ concentrations but not during low conditions [99]. Another signaling cascade involves CLE (CLAVATA3/ESR-RELATED) and its homologs (CLE1, -3, -4, -7), which interact with CLAVATA1 (CLV1) leucine-rich repeat receptor-like kinase to suppress lateral root growth under low NO_3_^−^ conditions [99]. Over-accumulation of mRNA levels of *CLE1, -3, -4*, and *-7* was also observed during N deficiency [100]. Lateral and primary root growth during N deficiency also relies on the phytohormone auxin. Repression of lateral root growth during N deficiency is also influenced by the ARF8/miR167 module, whereby the unavailability of N turns on miR167, which targets ARF8, causing failure of lateral root growth from the pericycle founder cells [101]. Similarly, *TAR2* (*TRYPTOPHAN AMINOTRANSFERASE-RELATED 2*), which is expressed in the pericycle, induces both primary and lateral root growth during mild N deficiency [102].

Under N limitations, the primary root elongates to absorb N from the deep soil [103]. It was previously reported that low N induces BRASSINOSTEROID ACTIVATED KINASE 1 (*BAK1*), a coreceptor that interacts with BRASSINOSTEROID-SIGNALING KINASE 3 (BSK3) to induce brassinosteroid signaling, thereby promoting primary root elongation [104]. During N deficiency, TEOSINTE BRANCHED1/CYCLOIDEA/PROLIFERATING CELL FACTOR1-20 (TCP20) and NODULE INTERCEPTION-LIKE PROTEIN (NLP6 and NLP7) interact with each other to form heterodimers, which then accumulate in the nucleus to negatively regulate CYCB1;1 (involved in the regulation of the G2/M cell-cycle). This ultimately helps increase root meristematic growth, giving rise to longer primary roots. ABSCISSIC ACID INSENSITIVE2 (ABI2), a protein phosphatase, was also found to be involved in the regulation of NRT1.1, leading to preferential root growth in high N compartments [105].

N deficiency also manipulates flowering time. Under limitations, *FNR1* causes an increase in NADPH/NADP^+^ and the ATP/AMP ratio, resulting in an increase in protein levels of AMPKα1, a subunit of the larger protein AMP-activated protein kinase (AMPK). This results in the failure of AMPK to induce phosphorylation of blue light receptor cryptochrome 1 (CRY1), which prevents its degradation and causes an early flowering phenotype [106,107]. Low NO_3_^−^ also causes an increase in the expression of GA biosynthesis genes, which again results in an early flowering phenotype [108]. Another response of plants under N starvation is increased anthocyanin accumulation. *NITROGEN LIMITATION ADAPTATION* (*NLA*) mutants failed to accumulate anthocyanin (cyanidins) but accumulated lignin instead [109]. Low N caused an increased survival of Arabidopsis seedlings; however, a mutant, *pap1*-*1*, did not exhibit the same result [110]. *PAP1-1* encodes for a MYB transcription factor (MYB75), which helps the plant to tolerate low N stress [110]. A metabolic shift to the phenylpropanoid pathway was found in plants under N-limiting conditions [109]. NO_3_^−^ limitations also result in a buildup of reactive oxygen species (ROS), like H_2_O_2_, which induces the expression of peroxidases in the root epidermis. Additionally, root hairs are thought to play a potential role in sensing nutrient concentrations [111,112]. Given the fact that ROS is an essential signaling component of signal transduction during abiotic stress, it might therefore be involved in the modification of the RSA during NO_3_^−^ deficiency [113].

### 2.3. Sensing and Signaling in Plants During N Deficiency

As described above, NO_3_^−^ is involved in numerous physiological processes, such as root architecture modifications, flowering, seed development, and shoot growth. Like P, it is also a signaling molecule, acting via two different signaling routes, local and systemic. Alterations in the local supply of NO_3_^−^ generally act at the cellular level, while systemic pathways sense the internal nutrient status and relay the signal to the shoots and other organs. Additionally, both the local and systemic pathways work together to alter the RSA [114].

Local perception of nutrient availability occurs at the roots and largely involves signals originating from changes in external nutrient concentrations. The most important NO_3_^−^ transporter, NRT1.1, which was first characterized in Arabidopsis, functions as a transceptor, possessing independent signal perception and nutrient transport functions [115,116]. NRT1.1 is also a dual affinity transporter, depending on the phosphorylation state of threonine (Thr) residue 101 for the switch [116,117]. Two protein kinases, CIPK8 and CIPK23 (CALCINEURIN B- LIKE-INTERACTING PROTEIN KINASE 23/SOS2-LIKE PROTEIN KINASE [PSK17]), are involved in the phosphorylation step, CIPK8 in low-affinity responses under high NO_3_^−^ conditions and CIPK23 during high-affinity responses under low NO_3_^−^ conditions [116,118]. NRT1.1 also presents a biphasic trend during the activation of PNRs, depending on the concentration of available NO_3_^−^. Phosphorylation switches NRT1.1 to a high-affinity transporter, activating *NRT2.1* and vice versa [116]. Unlike NO_3_^−^ deficient conditions, during which CALCIUM-SENSOR PROTEIN KINASES (CPK10, CPK30, and CPK32) transmit calcium signals downstream, no such signaling proteins have yet been identified that relay signals to the nucleus under N starvation [119]. NLP6 or NLP7 is the main regulator of NO_3_^−^ stress signaling, interacting with TCP20 in the nucleus through PB1, and inducing NO_3_^−^ starvation responses by binding to the NO_3_^−^ response cis-element (NRE) of NIR and NIA1 [120].

In the calcineurin B-like proteins (CBL) family, which sense changes in calcium levels by transiently interacting with CBL-interacting protein kinases (CIPKs), CBL7 reportedly plays a role in NO_3_^−^ starvation signaling. This protein is localized in the cytoplasm and nucleus, where it is expressed in high quantities. In young plants, *cbl7* mutants result in the downregulation of *NRT2.4* and *NRT2.5* [121].

One very important feature of plants is the ability to communicate the root nutrient status to the shoots and vice versa, allowing other pathways to be restructured and N transport and metabolism to be adjusted along with growth and development. ‘Split-root’ experiments refer to studies in which plant roots are divided into two halves and grown in different compartments. For example, in the presence and absence of N on opposite sides. Such experiments allow the effect of deficiency in one compartment on the other to be examined, helping reveal how N-related signals are transmitted from the deficient to the non-deficient site and how this influences subsequent responses [92]. NO_3_^−^ acts as a signal during root–shoot communication of N status, as demonstrated in NO_3_^−^ reductase-null mutants [122]. Meanwhile, levels of GNC (GATA transcription factors) were also found to increase in the shoots during N starvation of the roots, resulting in increased chlorophyll synthesis and sugar transport in lines overexpressing the transcription factor coding gene, thereby confirming root–shoot signal transduction under N starvation [123].

RNA-sequencing analyses also revealed that shoot N status influences root N uptake and root growth [124]. The most important players during the relay of root–shoot–root signals under N deficiency are root-derived, xylem mobile C-TERMINALLY ENCODED PEPTIDEs (CEPs), which are perceived by C-TERMINALLY ENCODED PEPTIDE RECEPTORS (CEPRs) in the shoots. This leads to the activation of two phloem mobile polypeptides, C-TERMINALLY ENCODED PEPTIDE DOWNSTREAM 1 (CEPD1) and CEPD2 (Class III Glutaredoxins), which traverse downwards to the roots [125,126,127]. CEPD1 and CEPD2 reportedly induce the upregulation of *NRT2.1* in the roots, resulting in N scavenging from NO_3_^−^ patches. The transmissible nature of root-derived CEPs was further revealed in grafting studies in which CEPs were found in the xylem sap [127]. Recent studies revealed that other genes also play a role in N uptake in deficient conditions. CEPD-LIKE 2 (CEPDL2) is a shoot-derived, phloem-mobile polypeptide that works in conjugation with CEPD1/2 to regulate plant N status [128]. *CEPDL2* is upregulated in the shoot in response to low shoot N and then travels downwards to the roots to facilitate nitrate uptake and N transport to the shoots.

Plants grown under low NO_3_^−^ conditions exhibit inhibited leaf growth and the exudation of cytokinins (CKs), such as zeatin and zeatin riboside, in the xylem, suggesting that NO_3_^−^ deprivation modifies shoot growth via long-distance signaling [129]. CK biosynthesis and signaling act as integrators of N supply and demand signals and help plants scavenge N from high-NO_3_^−^ patches under heterogeneous conditions [92]. For example, NO_3_^−^-activated CK plays a role in a signal transduction pathway that negatively regulates primary root growth in response to NO_3_^−^ [130]. The cytokinin biosynthesis gene *IPT3* is upregulated by NO_3_^−^ in Arabidopsis phloem tissue [130]. CK then subsequently induces the expression of glutaxarime genes *GRXS3/4/5/8*, and the GRXS3/4/5/8 proteins then act on roots to inhibit primary root growth [130].

**Figure 2:**
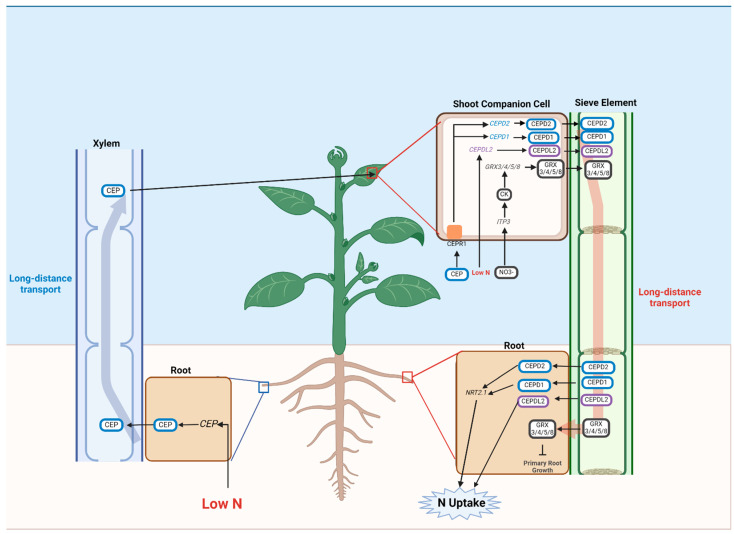
Systemic responses to low N in plants. CEP: -terminally encoded peptide; CEPD1: cep downstream 1; NRT2.1: high affinity nitrate transporter 2.1; GRX3/4/5/8: glutaredoxin 3/4/5/8; CEPDL2: cepd-like 2; IPT3: adenylate isopentenyltransferase 3; CK: Cytokinin. This image was generated using biorender.com.

In another split-root study, disruption of CK biosynthesis in the shoots was found to result in a lack of trans-Zeatin (tZ) accumulation, attenuating the nutrient foraging responses of the roots under heterogeneous soil NO_3_^−^ conditions. More importantly, CK biosynthesis and transport also affected glutamate/glutamine metabolism [131]. The systemic signaling in response to N deficiency is summarized in Figure 2.

### 2.4. Genetic Responses of Plants During N Starvation

N starvation results in a number of changes to the genetic landscape of a plant. NO_3_^−^ itself acts as a signaling molecule, inducing a series of transcriptional modifications known as Primary Nitrate Responses (PNRs), which are predominantly under genetic control and are unaffected by protein inhibitors [132]. NLP and NIGT1/HHO transcription factors are also central to N starvation-induced gene expression patterns, with NLP upregulating the expression of NO_3_^−^ responsive genes and NIGT1/HHO repressing expression [133]. Meanwhile, under low substrate availability, high-affinity transporters such as NRT2 and AMT1 are more active [134,135]. Notable genes that are induced during this response include NO_3_^−^ transporters (*NRT1.1*/*NPF6.3*, *NRT2.1* and *NRT2.2*), nitrate reductase (*NIA1* and *NIA2*) and the nitrite reductase (*NIR*) [132]. Arabidopsis *miR169* is also downregulated during N deficiency, which fails to induce the downregulation of *NRT1.1* and *NRT2.1* [136].

Like most biochemical pathways, genetic reprogramming is induced by several transcription factors. ANR1 was the first transcription factor to be identified as a MADS-Box, mutations of which prevent lateral root development under NO_3_^−^ sufficient conditions [137]. Other transcription factors, such as GNC, are also induced during N starvation [123]. Moreover, just-in-time (JIT) analysis of time-series transcriptome data revealed that CYTOKININ RESPONSE FACTOR4 (CRF4) is an upstream transcription factor that mediates NO_3_^−^ assimilation during N starvation, with eight of its members functional in the shoots (CRF1–6, CRF10, and CRF11) and three (CRF3, CRF4, and CRF11) in the roots [138]. Members of the nuclear factor Y subunit A transcription factors are also positively induced during N starvation. For example, transcript levels of *NUCLEAR FACTOR-Y* (*NFYA3*, *NFYA5*, and *NFYA8*) were found to accumulate in the roots, with *NFYA1*, *NFYA9*, and *NFYA10* accumulating in the shoots and *NFYA2* accumulation equally in both the roots and shoots [136]. Meanwhile, other transcription factors, such as LATERAL ORGAN BOUNDARIES DOMAIN (*LBD37* and *LBD39*), *HHO1*, and *HRS1*, which are also essential for NO_3_^−^ signaling and RSA modulations, were found to be downregulated [139,140,141]. HRS1 was also found to repress several NO_3_^−^ transporters, such as *NRT2.4* and *NRT2.5*, during NO_3_^−^ starvation, thereby helping the plant scavenge NO_3_^−^ [142]. The role of epigenetic modifications during N starvation is relatively unknown. However, in a study on rice, low N was found to induce a heritable methylation pattern and increased tolerance in subsequent progenies [143].

A major component of success in agricultural production is the application of chemical fertilizers, e.g., N and P [144]. However, although insufficient N and P are common in the field, very few studies have investigated the responses of plants to the combined deficiencies. To enhance crop yield and improve nutrient use efficiency (NUE), it is therefore important to understand how these two nutrients interact with each other in terms of signaling, metabolism, and physiology [43].

## 3. Nitrogen and Phosphorus

### 3.1. Morphological and Biochemical Processes Controlled by N-P Interactions

N and P interactions control a number of biochemical processes, one of which is the colonization of arbuscular mycorrhizal (AM) fungi. Deficiency results in increased colonization of these fungi, controlled at a systemic level with the induction of two Pi transporter genes (*MtBCP1* and *MtPT4*) in plants under combined N and P deficiency but not under respective low levels. Meanwhile, accumulation of high amounts of Pi was also observed in *Medicago trunculata* plants under combined deficiency compared to respective N then Pi deficiency, suggesting that N is the deciding factor regulating Pi accumulation via AM fungi [145]. The interaction between these two nutrients was also demonstrated in tomato plants, where an increase in Pi led to a steep increase in leaf Pi concentrations, while an increase in N alone resulted in a gradual increase, with leveling off both nutrients at high concentrations, resulting in reduced N accumulation and changes in relative growth rates (RGR) [146]. Most of the N in plants is utilized for energy metabolism, while a very low amount is used in structural constituents, and vice versa with the P pool. However, because of the interdependency of metabolic and structural processes, these two nutrients are highly correlated. For example, improved metabolism in plants with the addition of N also affects how the plant performs in structural processes such as the partitioning of proteins, carbohydrates, and metabolites for the development of cellular organelles and subsequent growth [146]. Although P plays a more direct role in RGR alterations, N is thought to be as important by these interactions. P deficiency also results in a decrease in soluble sugars, which reduces the pool of carbohydrates available for assimilation, thereby reducing N uptake by the plant [146]. A correlation was also found between the decrease in N accumulation under low P conditions and reductions in cytokinin levels, nitrate reductase activity, and net protein synthesis [147]. Reduced cytokinin levels compromise the root–shoot movement of N, leading to an overaccumulation in the roots. This buildup then potentially leads to negative feedback inhibition of N uptake, causing a net decrease in N levels [146]. Meanwhile, P limitations also cause higher export of sugars from the shoot to the roots, as shown by reductions in starch concentrations. For example, in tomato, a decrease in soluble sugars reduced the pool of carbohydrates available for N assimilation, consequently reducing N uptake by the plant [146]. Similarly, in soybean, P limitation was found to result in an accumulation of N^15^ labeling in the roots and a reduction in ^15^NO_3_^−^ uptake [147]. In addition, asparagine levels in the roots and stems also increased [147].

Acid phosphatases (APs) are induced by Pi starvation and help scavenge organic forms of Pi. A previous study revealed that N is essential for the induction of *AtACP5* in the mature zone of the roots, while combined deficiency with P led to the inhibition of P rescue mechanisms [148]. Another study revealed that *AtPT1*, a Pi transporter, also shows a similar trend, suggesting that N is essential for Pi rescue and transport gene induction [149]. Importantly, with increasing concentrations of NO_3_^−^, expression of *AtPT1* was found to span the entire root system from just a minute root tip [149]. N also controls meristematic activity, with differential responses in the presence and absence of Pi, which is further suggestive of interaction [149]. Cells expressing cyclophilin B were unchanged by Pi limitation; however, when both nutrients were limited, the *CycB* gene was repressed, suggesting that both nutrients are necessary for mitotic activity in plant cells [149].

N and P combined are also involved in the modulation of flowering times in Arabidopsis. This could be due to the regulation of the accumulation of N and P during the presence of either of the nutrients. This interplay in their accumulation is controlled by an unknown NO_3_^−^ and NLA-regulated pathway [117,150]. Under low NO_3_^−^ and high Pi conditions, flowering was earlier when the floral induction genes (floral induction genes (e.g., *FLOWERING LOCUS T (FT)*, *LEAFY* (*LFY*) and *APETALA1* (*AP1*)) were activated. However, in the case of low Pi and high NO_3_^−^ conditions, floral suppressor genes (*FLOWERING LOCUS C* (*FLC*)) were activated [150,151,152]. Earlier studies also suggest prolonged vegetative growth with the application of NO_3_^−^, while in Arabidopsis, limitation of both nutrients was found to have a significant effect on senescence, suggesting an interaction between NO_3_^−^ and Pi signaling pathways during flowering [150,151].

### 3.2. Main Players in the N-Pi Interaction Cascade

N has been shown to play an overlapping and vital role in Pi starvation signaling. On a global scale, this was experimentally shown in Mongolian grasslands, where the addition of Pi alone in the absence of N caused no major changes in the plant nutrient composition or stoichiometries, suggesting that N availability is crucial in determining P uptake and assimilation [153]. The availability of N was also found to play an essential role in the accumulation of nitrogenous compounds in source leaves during Pi limitation in young barley plants [154]. Availability largely determined which compound accumulated—nitrates and amino acids in high N plants and amino acids and proteins in low N plants—probably due to the slowing down of phloem exports from the sink to source leaves. In line with this, deficiency of both N and P was found to cause a reduction in the exportation of amino acids through the phloem [154]. Meanwhile, during both N and P starvation, plants prioritize N uptake, which helps activate PSRs that are shut off when N is lacking. N provision also downregulates PHO2, a repressor of PSRs [155]. Under low NO_3_^−^ conditions, PHO2 causes a reduction in PHR1 accumulation, while repression of PHO2 is thought to be controlled by NRT1.1 [155]. A de-repression of PSI genes (IPS1, PHT1-1, and SPX1) in *pho2* mutants was also reported, suggesting that NO_3_^−^ controls PSRs in a *PHO2*-dependent manner [155]. However, transcript accumulation of *miRNA399* was found to increase when plants were supplied with NO_3_^−^. PHO2 was also found to control protein levels of NRT1.1, suggesting a circular loop in which PHO2 is located both upstream and downstream of the PSR cascade, adding complexity to the interlinked nature of N and P starvation signaling pathways [76,156].

NO_3_^−^ and Pi function as individual signaling molecules, each possessing elaborate signaling pathways under low nutrient conditions. NLPs in the NO_3_^−^ signaling pathway and the MYB2-type CC transcription factor PHR1 are central to these pathways, activating the expression of numerous NO_3_^−^ (*NRT2.1*) and Pi-responsive genes (e.g., *PHTs*) [157]. However, both nutrients interact at a molecular level during deficiencies [133]. One of the most striking physiological features of this interaction occurs during root development. Pi starvation in the presence of NO_3_^−^ results in an arrest of primary root development. This cascade works via a GARP-type transcription factor known as NIGT1/HRS1, which further coordinates with HHO1 to repress downstream genes involved in root development [139]. NIGT1/HRS1 is activated by NRT1.1 and the NLP7 cascade, which depends on the presence of NO_3_^−^. NIGT1/HRS1 also represses the cis-elements involved in root development, such as TCP (TEOSINTE BRANCHED1, CYCLOIDEA, PCF), as well as the repressors of PSI genes in the presence of NO_3_^−^ [139,158]. More recently, *OsNIGT1* was reported to directly bind to promoters of Pi starvation signaling genes such as *IPS1*, *miR827*, and *SPX2* to modulate PSRs [159].

To determine the missing link between NO_3_^−^ and Pi signaling pathways, the role of NO_3_^−^ in mediating the interaction between SPX4 protein and NRT1.1 during the degradation of SPX4 was examined. SPX4 is also a repressor of PSI and acts by binding to PHR2 [160]. Accordingly, irrespective of the Pi concentration, NRT1.1 was found to use NBIP1, a component of 26S proteasomal ubiquitination machinery, to degrade SPX4 in the presence of NO_3_^−^. This interaction causes PHR2 to enter the nucleus, where it activates a number of PSI genes, such as *IPS1*, *PT2*, and *IPS2*. Importantly, even NLP was found to be under the control of SPX4, further integrating both pathways [160]. PHR1 was also found to control NO_3_^−^ signaling by binding to the promoters of NIGT1, in turn repressing *NRT2.1* and reducing the uptake of NO_3_^−^ [98].

### 3.3. Other Players in the N-Pi Interaction

Cytokinin is closely associated with both N and P signaling modules [149,161]. An antagonistic relationship between cytokinin and NO_3_^−^ was also observed, resulting in the repression of *AtPT1* and *AtACP5* (two PSI genes), regardless of the presence of Pi [162]. NO_3_^−^ also induces the expression of several cytokinin biosynthesis genes, such as *AtIPT1*. However, the antagonistic relationship suggests that the control of NO_3_^−^ over cytokinin biosynthesis, and thereafter PSR, are independent of each other [162]. Furthermore, when both nutrients were unavailable, cytokinin concentrated *AtPT1* and *AtACP5* in the root hair zone, suggesting that N is an important factor controlling Pi starvation responses, with partial mediation via cytokinins [149].

Mobile micro-RNAs such as *miR827* are also thought to play a role in controlling Pi and NO_3_^−^ signaling, both individually and in conjunction [163]. Under Pi starvation, *miR827* targets SPX-MSF1, a negative regulator of Pi signaling [163]. *NLA* and *PHO2* genes are also negative regulators of Pi uptake, requiring the presence of NO_3_^−^ to carry out this role [150]. In the absence of NO_3_^−^, *NLA* and *PHO2* fail to suppress Pi uptake, leading to Pi toxicity coupled with high anthocyanin levels. Meanwhile, *PHF1* and *PHT1.1* further suppress *NLA* in *nla* and *pho2* plants, having reverted back to normal Pi concentrations. Both *miR827* and *miR399* help suppress *NLA* and *PHO2* under optimal Pi levels, preventing the overaccumulation of Pi [150]. These findings add to our overall understanding of the role of microRNAs in the close interaction between NO_3_^−^ and Pi signaling pathways [150].

### 3.4. Long-Distance Signaling of N-P Interaction

Long-distance or systemic signaling takes place in most flowering plants, controlling a host of whole-plant responses, as experimentally demonstrated in split-root experiments [92]. Cytokinins are thought to function as a messenger under nutrient deprivation. Two forms of cytokinin, trans-Zeatin (tZ) and N6-(D2-isopentenyl adenine (iP), are active in the roots and shoots, and the tZ type help promote shoot growth under high NO_3_^−^ conditions [150,164]. In a series of reports, it was found that cytokinin also controls the activity of Pi transporters through the ARABIDOPSIS HISTIDINE KINASE 4/CYTOKININ RESPONSE 1/ WOODEN LEG (AHK4/CRE1/WOL) and AHK3 modules [64]. Since cytokinin biosynthesis is closely linked to the presence of NO_3_^−^, they are therefore thought to act as messengers of NO_3_^−^ availability and subsequent uptake of Pi [161].

**Figure 3:**
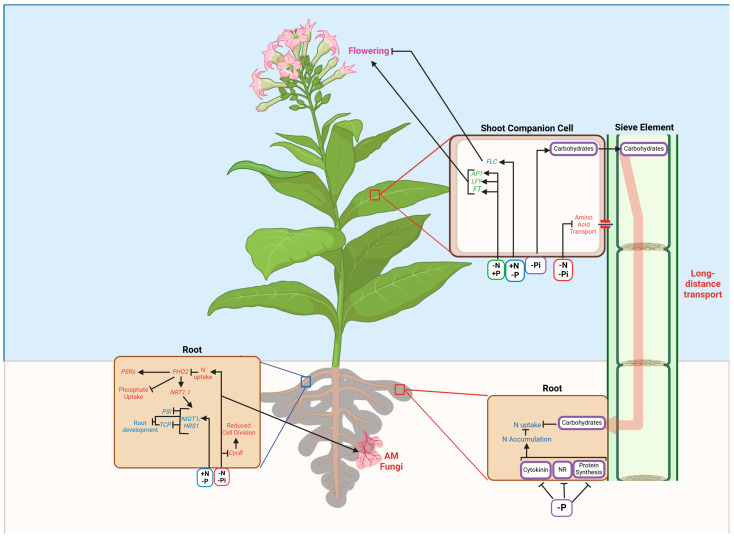
Molecular responses to combined N and P starvation in plants. NIG1/HRS1: hypersensitivity to low Pi-elicited primary root shortening 1; NRT1.1: nitrate transporter 1.1; CycB: cyclin-dependent protein kinase B; AP1: apetala1; LFY: leafy; FT: flowering locus T; FLC: flowering locus C. The image was generated using biorender.com.

RNA-sequencing analyses suggest that shoot N status influences root growth and root N uptake [124]. The most important player in the relay of the root–shoot–root signals under N deficiency are CEPs, which are perceived by CEPRs, a leucine-rich repeat-receptor kinases (LRR-RKs), leading to the activation of two phloem mobile polypeptides, CEPD and CEPD2 (Class III Glutaredoxins), which traverse downwards to the roots [125,126,127]. CEPD1 is thought to cause upregulation of *NRT2.1* in the roots, resulting in scavenging of N from NO_3_^−^ patches. The transmissible nature of root-derived CEPs was also revealed in grafting studies, with identification in the xylem sap [127]. Overall, these findings suggest that CEPDs potentially act as messengers of N status in the control Pi signaling. The systemic signaling in response to N and P deficiency is summarized in Figure 3.

## 4. Practical Application of N and P Starvation Genes in Crop Improvement

Functional characterization of genes induced during nutrient deprivation helps in the identification of candidates that can be overexpressed or knocked down for improvement of nutrient use efficiencies. Individual applications of genes have been performed for each macronutrient at both local and systemic levels. For example, overexpression of a MADS transcription factor, *TaMADS2-3D*, led to enhanced Pi uptake by modulating ROS homeostasis in wheat roots [165]. Similarly, overexpression of *GmWRKY46* in soybeans led to increased tolerance to Pi starvation and lateral root development [166]. Combined activation of 2 transporters; *OsAMT1;2* and *OsGOGAT1*, optimized ammonium uptake, N remobilization, and enhanced tolerance to N limitation [167]. On the other hand, there have been reports of improving concurrent utilization of N and P by altering a single gene. Simultaneous N, P, and Fe uptake was boosted by the overexpression of a pH-sensitive plasma membrane-bound transporter *OsNRT2.3* [168]. Functional characterization of a rice mutant, 88n (mutation in *OsbZIP1*) in rice revealed activation of Pi transport genes in roots and nitrate utilization [169].

In addition, several genes that regulate systemic signaling also have the potential for improvement. For instance, miR399 plays a role in root–shoot signaling [70]. Overexpression of miR399 leads to overaccumulation of P in Arabidopsis [170]. However, titratable overexpression strategies with estradiol can be employed to fine-tune Pi uptake in crop plants [171]. Similar approaches to overexpress CEPD can improve the systemic signaling during N starvation and therefore improve crop yields in N-deprived conditions [127]. Additionally, previous research identified several genes in the phloem that are altered, including *Susy2* and *PAP15*. Both of these genes serve as excellent targets for rewiring long-distance signaling to improve P use efficiency [76]. Finally, overexpression of genes mediating starvation responses of both N and P, such as the GARP family of transcription factors, can be utilized to simultaneously improve N and P use efficiencies in nutrient-deprived agricultural ecosystems [172].

## 5. Future Perspectives

Among plant organs, roots and shoots are the primary centers for signal perception and transduction. Despite the rapid progress in understanding local signaling during either single or combined nutrient starvation in plants, more emphasis needs to be placed on the systemic signaling mechanisms. Understanding how these organs perceive and integrate signals and how they communicate with each other under mineral deficiency will provide insights into reinforcing abiotic stress-resistance genes in crop plants. Phloem tissue is crucial for the systemic signaling of nutrient starvation. Accurate identification of the molecular components relies on authentic methods for collecting phloem from plants. However, most of the currently existing methods, such as aphid stylectomy and EDTA-facilitated extraction, are problematic. Different from most other plants, extracting pure vascular tissues from *Plantago* is easy [76]. Recently, the genome of *Plantago* has been sequenced and a transformation system has also been developed. Future researchers can adopt this species to identify genes and pathways associated with the vascular tissues in response to individual or combined mineral-deficient growth conditions. The understanding of the mechanisms from this new model species will not only improve our understanding of basic plant phsysiology but will also help implement strategies to increase the mineral uptake and use efficiency in other crop plants.
